# Immune checkpoint inhibitors for metastatic uveal melanoma: a meta-analysis

**DOI:** 10.1038/s41598-024-55675-5

**Published:** 2024-04-03

**Authors:** Kayoko Yamada, Masaki Takeuchi, Takeshi Fukumoto, Minako Suzuki, Ai Kato, Yuki Mizuki, Norihiro Yamada, Takeshi Kaneko, Nobuhisa Mizuki, Nobuyuki Horita

**Affiliations:** 1 Department of Ophthalmology and Visual Science, Yokohama City University Graduate School of Medicine, 3-9 Fukuura, Kanazawa-ku, Yokohama, 236-0004 Japan; 2 Division of Dermatology, Department of Internal Related, Kobe University Graduate School of Medicine, Kobe, Japan; 3 Department of Pulmonology, Yokohama City University Graduate School of Medicine, Yokohama, Japan; 4 Chemotherapy Center, Yokohama City University Hospital, 3-9 Fukuura, Kanazawa-ku, Yokohama, 236-0004 Japan

**Keywords:** Cancer immunotherapy, Eye cancer

## Abstract

Several studies have evaluated immune checkpoint inhibitors (ICIs) for metastatic uveal melanoma; however, the efficacy of ICIs in the previous studies varied greatly. In this systematic review, we searched for prospective or retrospective studies on single or dual-ICIs for metastatic uveal melanoma treatment. A random-effect model meta-analysis with generic inverse-variance was conducted, and 36 articles representing 41 cohorts of 1414 patients with metastatic uveal melanoma were included. The pooled outcomes were as follows: objective response rate (ORR) was 5.6% (95% confidence interval [95%CI] 3.7–7.5%; I^2^, 36%), disease control rate (DCR) was 32.5% (95% CI 27.2–37.7%; I^2^, 73%), median progression-free survival was 2.8 months (95% CI 2.7–2.9 months; I^2^, 26%), and median overall survival (OS) was 11.2 months (95% CI 9.6–13.2 months; I^2^, 74%). Compared to single-agent ICI, dual ICI led to better ORR (single-agent: 3.4% [95% CI 1.8–5.1]; dual-agent: 12.4% [95% CI 8.0–16.9]; P < 0.001), DCR (single-agent: 29.3%, [95% CI 23.4–35.2]; dual-agent: 44.3% [95% CI 31.7–56.8]; P = 0.03), and OS (single-agent: 9.8 months [95% CI 8.0–12.2]; dual-agent: 16.3 months [95% CI 13.5–19.7]; P < 0.001). Our analysis provided treatment outcomes as described above. Dual-ICIs appear better than single-agent ICIs for the treatment of metastatic uveal melanoma.

## Introduction

Uveal melanoma is a rare subtype of melanoma; nevertheless, it is the most common primary intraocular malignancy in adults^1–3^. The prognosis for patients with advanced uveal melanoma remains poor^4^. A systematic review of 78 articles published between 1980 and 2017 presents the median overall survival (OS) of 12.8 months, although many of these studies were conducted before ICI became available^5^. Uveal melanoma is considered a tumor distinct from cutaneous melanoma based on clinical and genetic heterogeneities^6,7^. Particularly, uveal melanoma is characterized by few driver mutations and rare passenger mutations, which has made all kinase inhibitors’ reporting ineffective^8–10^. Therefore, patients with metastatic uveal melanoma have often been treated with immune checkpoint inhibitor (ICI) regimens despite the lack of robust evidence. Tebentafusp, a bispecific T-cell engager, demonstrated the first OS benefit for metastatic melanoma in a HLA-A*02:01-positive patient^11^. However, the treatment strategy for metastatic uveal melanoma in HLA-A*02:01-negative patients remains to be established. In addition, a recently featured oncological topic is the choice between single-agent and combined ICI for cancer treatments^12–16^. Particularly, combining nivolumab and ipilimumab has been frequently assessed in trials, and dual ICI regimens, rather than monotherapies, are the first choice for cutaneous melanoma^17^. However, whether dual ICI therapy is the more plausible treatment for metastatic uveal melanoma than single-agent ICI remains controversial.

To date, several clinical trials and observational studies have evaluated ICIs for metastatic uveal melanoma treatment, and ICI efficacy is of considerable interest to all physicians treating patients with metastatic uveal melanoma. However, ICI efficacy, as reported in previous studies, varies greatly. Therefore, we designed a systematic review and meta-analysis to provide novel insights concerning the objective response rate (ORR), disease control rate (DCR), progression-free survival (PFS), and OS among patients with metastatic uveal melanoma treated with ICIs with a particular interest in the possible difference between single-agent and dual ICI regimens.

## Methods

### Protocol registration

The protocol of this systematic review was registered as UMIN000047431 on the University Hospital Medical Information Network (UMIN) Registration website on April 7, 2022 (available at https://www.umin.ac.jp/ctr/index.htm). Institutional review board approval and informed consent were not mandatory because the study involved aggregated data. All methods were performed in accordance with the relevant guidelines and regulations.

### Study search

Two investigators (K.Y. and M.T.) systematically and independently searched for eligible articles from electronic databases such as PubMed, Web of Science, Cochrane Central Register of Controlled Trials, and EMBASE until April 7, 2022. The following search formula was used for PubMed: (immune checkpoint inhibitor OR immune checkpoint inhibitors OR ICI OR ICB OR immune checkpoint blockade OR immune checkpoint blockades OR nivolumab OR pembrolizumab OR spartalizumab OR cemiplimab OR avelumab OR atezolizumab OR durvalumb OR ipilimumab OR tremelimumab OR camrelizumab OR sintilimab OR sugemalimab) AND (melanoma) AND (ocular OR eye OR ophthalmological OR intraocular OR uveal OR uvea OR iridal OR iris OR ciliary OR choroidal OR choroid OR chorioidea OR ciliochoroidal). The search terms for the other databases are listed in Supplementary Table 1. Two investigators (K.Y. and M.T.) independently performed additional searches manually. In addition, we attempted to have email communication on a few articles.

### Study selection criteria

This systematic review included prospective or retrospective studies reported in English that provided data on at least one of the four outcomes of ICIs for patients with metastatic uveal melanoma. Case reports, studies with fewer than five patients, and conference abstracts were excluded. In addition, the duplicate use of the same data was not permitted.

### Patient selection criteria

This study focused on patients with metastatic uveal melanoma Studies focused on non-uveal ocular melanomas, such as iridial and ciliary melanomas, were excluded. However, when a study simply dealt with ocular melanoma, it was not excluded because most ocular melanomas were expected to be uveal melanoma. The metastatic organ of uveal melanoma has not been investigated.

### Treatment

Anti-programmed cell death protein 1 (PD-1), anti-programmed death ligand 1 (PD-L1), and anti-cytotoxic T-lymphocyte-associated protein 4 (CTLA-4) antibodies were used. Both the single-agent and dual-ICI regimens were accepted. Even when the drug’s name was unclear, one article was included in our study. Subgroup analyses separately evaluated the patients who received single-agent therapy and combination therapies. When a study collectively analyzed patients with single- and double-ICI regimens, it was grouped as a “mixed” category. Studies/data on patients receiving combination therapy with non-ICI agents, adjuvant therapy, and neo-adjuvant therapy were excluded.

### Outcome

The co-primary endpoints of our analysis were single-arm ORR, DCR, median PFS, and median OS. ORR is the sum of complete and partial responses, and DCR is the sum of complete response, partial response, and stable disease^18^.

### Data extraction

Two review authors (K.Y. and M.T.) independently extracted the research data from the original reports using data extraction form (Supplementary Table 2). When an article described clearly different regimens, data of such a study was subdivided into two or more cohorts.

### Study quality assessment

The quality of the included studies was assessed using the Newcastle–Ottawa Scale for cohort studies, wherein a maximum score of eight points indicated the best quality^19^.

### Statistics

The main meta-analyses were performed by applying the random-effects model generic inverse variance method^20^. In addition, sensitivity meta-analysis based on the fixed-effect model was conducted. The binary outcomes, namely ORR and DCR, were pooled after the standard error (SE) was estimated using the Wilson score interval^21^. ORR is a measure of the proportion of patients who experience partial or complete response on the response evaluation criteria in solid tumors^18^. Similarly, DCR indicates the proportion of cases with stable disease, partial response, and complete response. Once the median survival time and 95% confidence interval (CI) were logarithmically transformed, the SE for survival data was calculated assuming a normal distribution using the following formula: SE = (log(upper limit of 95% CI) log (lower limit of 95% CI)) /2/1.96^22^. When necessary, the 95% CI of survival data was obtained as the time point at which the upper and lower 95% CI of survival proportions crossed 50% of survival. The heterogeneity evaluated with the I^2^ statistics was interpreted in a standard manner^23^. In short, 0–40% might not be important, 30%–60% may represent moderate heterogeneity, 50–90% may represent substantial heterogeneity, and 75–100% indicates considerable heterogeneity^20^.

All analyses were performed using Review Manager ver 5.4.1 (Cochrane Collaboration, Oxford, UK). The figures illustrated using Review Manager were adjusted as necessary.

## RESULTS

### Study search

Of 808 non-duplicated articles that met the preliminary criteria, 369 and 403 were excluded through title/abstract screening and full article reading, respectively. We identified 36 eligible articles representing 41 single-arm-level cohorts (Supplementary Fig. 1 and Table 1)^24–59^. The total number of patients in all the studies was 1414 (Table 1). No randomized controlled trial was identified. The number of patients in a cohort treated with ICIs was 5–125 (Table 1). The mean or median age of the patients in each study was 42–67 years. The Newcastle–Ottawa Scale score was 5–7 points, suggesting that most studies were of reasonable quality (Table 1).

**Table 1:** Characteristics of included studies.

Study	Country	Design	NOS	N	Age (y)	Men (%)	Regimen
Ahmad 2015^24^	UK	Retro	6	15	NA	NA	Ipi 3 mg/kg q3w
Alexander 2014^25^	Australia	Retro	6	11	NA	NA	Ipi 3 mg/kg q3w
Algazi 2016^26^	USA	Retro	6	58	62	NA	Pemb, miv, atez
Bender 2017^27^	Germany	Retro	5	15	62	33	Pemb 2 mg/kg q3w, Niv 3 mg/kg q2w
Bol 2019 A^28^	Denmark	Retro	7	24	NA	NA	Ipi
Bol 2019 B^28^	Denmark	Retro	7	43	NA	NA	Pem
Bol 2019 C^28^	Denmark	Retro	7	19	NA	NA	Ipi + Niv
Danielli 2012^29^	Italy	Pro	6	13	57	62	Ipi 10 mg/kg q3w + Ipi 10 mg/kg q12w
Heppt 2017 A^30^	Germany	Retro	6	54	NA	54	Pemb 2 mg/kg q3w
Heppt 2017 B^30^	Germany	Retro	6	32	NA	59	Niv 3 mg/kg q2w
Heppt 2017 C^30^	Germany	Retro	6	15	NA	67	Pem + Niv
Jansen 2020^31^	Belgium	Pro	6	9	61	22	Pemb 2 mg/kg q3w
Johnson 2019^32^	USA	P2	6	5	63	NA	Pemb
Joshua 2015^33^	Canada	P2	6	11	58	64	Trem at 15 mg/kg q90d
Karydis 2016^34^	UK	Retro	6	25	58	52	Pemb 2 mg/kg q3w
Keilholz 2019^35^	Germany	P1b	6	16	NA	NA	Avel 10 mg/kg q2w
Kelderman 2013^36^	Netherlands	Pro	6	22	54	45	Ipi 3 mg/kg q3w
Kelly 2021^37^	Canada	Retro	6	71	64	47	anti-PD1, anti-PD1 + anti-CTLA4
Khattak 2013^38^	UK	Retro	6	5	42	20	Ipi 3 mg/kg q3w
Kirchberger 2018^39^	Germany	Retro	6	9	66	67	Ipi 1 mg/kg q3w + Pemb 2 mg/kg q3w
Klemen 2020^40^	USA	Retro	6	30	66	57	Ipi, Niv, Pemb, Niv + Atez, Pemb + Atez
Koch 2022 A^41^	Germany	Retro	6	52	64	52	Ipi, Niv, Pemb, dual ICI
Koch 2022 B ^41^	Germany	Retro	6	125	67	50	Ipi, Niv, Pemb
Kooij 2017^42^	Netherlands	Retro	6	17	NA	30	Pemb 2 mg/kg q3w, Niv 3 mg/kg q2w
Kottschade 2016^43^	USA	Pro	5	10	65	30	Pemb 2 mg/kg q3w
Luke 2013^44^	USA	Retro	6	39	61	59	Ipi 3 mg/kg, Ipi 10 mg/kg
Maio 2013^45^	Italy	Pro	6	83	62	47	Ipi 3 mg/kg q3w
Mignard 2018^46^	France	Retro	7	100	65	43	Ipi 3 mg/kg q3w, Niv 3 mg/kg q2w, Pemb 2 mg/kg q3w
Moser 2015^47^	USA	Retro	6	23	NA	NA	Ipi
Najjar 2020^48^	USA	Retro	6	89	53	53	Ipi + Niv
Namikawa 2020^49^	Japan	Retro	6	14	59	79	Niv 3 mg/kg q2w, Niv 2 mg/kg q3w
Nathan 2019^50^	UK	P2	6	103	63	56	Niv 3 mg/kg q2w
Pelster 2021^51^	USA	P2	6	35	62	34	Niv 1 mg/kg + Ipi 3 mg/kg
Piulats 2021^52^	Spain	P2	6	52	59	56	Niv 1 mg/kg q3w + Ipi 3 mg/kg q3w, followed by Niv 3 mg/kg q2w
Rossi 2019^53^	Italy	Pro	6	17	64	53	Pemb 2 mg/kg q3w
Sander 2021^54^	Canada	Retro	6	37	59	57	(Pemb 2 mg/kg q3w, Niv 3 mg/kg q2w, Ipi 3 mg/kg + Niv1 mg/kg q4w) followed by Niv 3 mg/kg q2w
Tacar 2021^55^	Turkey	Retro	6	16	60	41	Niv 3 mg/kg q2w
Wiater 2013^56^	Poland	Retro	6	9	49	58	Ipi 3 mg/kg q3w
Xu 2019^57^	USA	Retro	7	18	63	52	Ipi, Niv, Pemb
Yasar 2020^58^	Turkey	Retro	6	20	NA	NA	Ipi
Zimmer 2015^59^	Germany	P2	6	53	67	43	Ipi 3 mg/kg q3w

A/B/C after the study author/year represents the cohorts in each study.

*N* patients in a cohort, Age mean or median age, *NA* not available. *Pro* prospective, *NOS* Newcastle–Ottawa quality assessment scale for cohort studies, Retro retrospective, *P2* Phase II trial, *ICI* immune checkpoint inhibitor, *Niv* nivolumab, *Pemb* pembrolizumab, *Ipi* ipilimumab, *Atez* atezolizumab, *Trem* tremelimumab, *Avel* avelumab, *q* every (quaque), *w* week, *d* day.

Of the 41 cohorts, 28 adopted retrospective studies and 7 were derived from phase I or II trials. Patients in 31 cohorts were treated with ICI monotherapy, including ipilimumab (n = 12), nivolumab (n = 4), pembrolizumab (n = 7), tremelimumab (n = 1), or avelumab (n = 1). Each of the six cohorts collectively assessed patients treated with different single-agent ICIs. In contrast, those in six cohorts were treated with a dual ICI regimen, PD-1 (nivolumab or pembrolizumab) plus CTLA-4 (ipilimumab). Four articles collectively presented data on single-agent ICI and dual ICIs.

### Objective response rate

**Figure 1:**
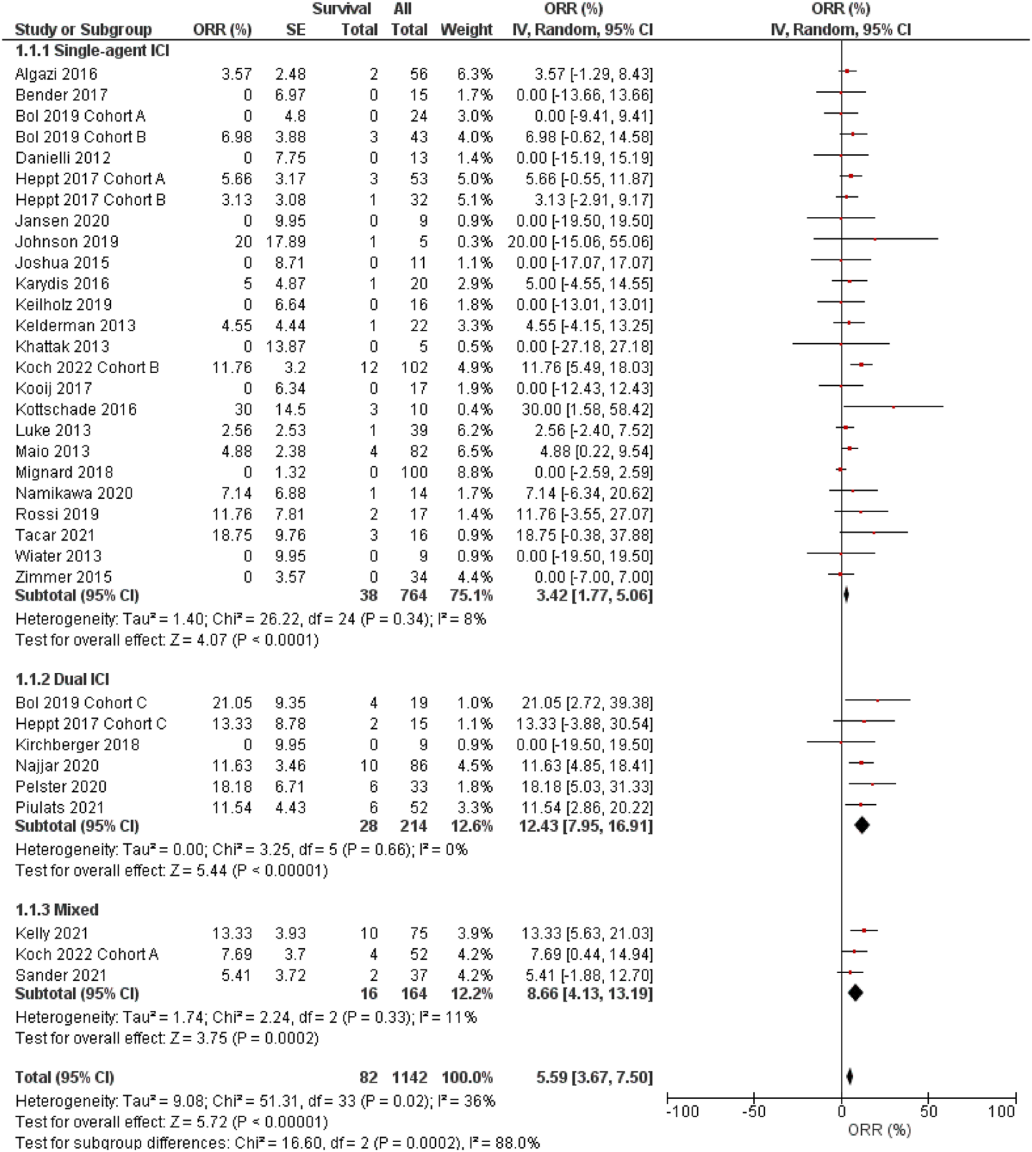
Forest plots for objective response rate (ORR, %) (random-effect model). *IV* inverse variance, *SE* standard error, *CI* confidence interval, *ICI* immune checkpoint inhibitor, mixed, a study that collectively analyzed patients with single- and double-ICI regimens. Subgroup comparison single- versus dual-agent ICIs, P < 0.001.

Random-model meta-analysis using the generic inverse variance method suggested a pooled ORR with any ICI regimen of 5.6% (95% CI 3.7–7.5%; I^2^ = 36%, P for heterogeneity = 0.02; Fig. 1). Despite the low ORR by the single-agent ICI (3.4%, 95% CI 1.8–5.1; I^2^ = 8%, P for heterogeneity = 0.34), that by dual-ICI (12.4%, 95% CI 8.0–16.9; I^2^ = 0%, P for heterogeneity = 0.66) achieved a higher ORR (subgroup comparison single-agent versus dual ICIs, P < 0.001).

### Disease control rate

**Figure 2:**
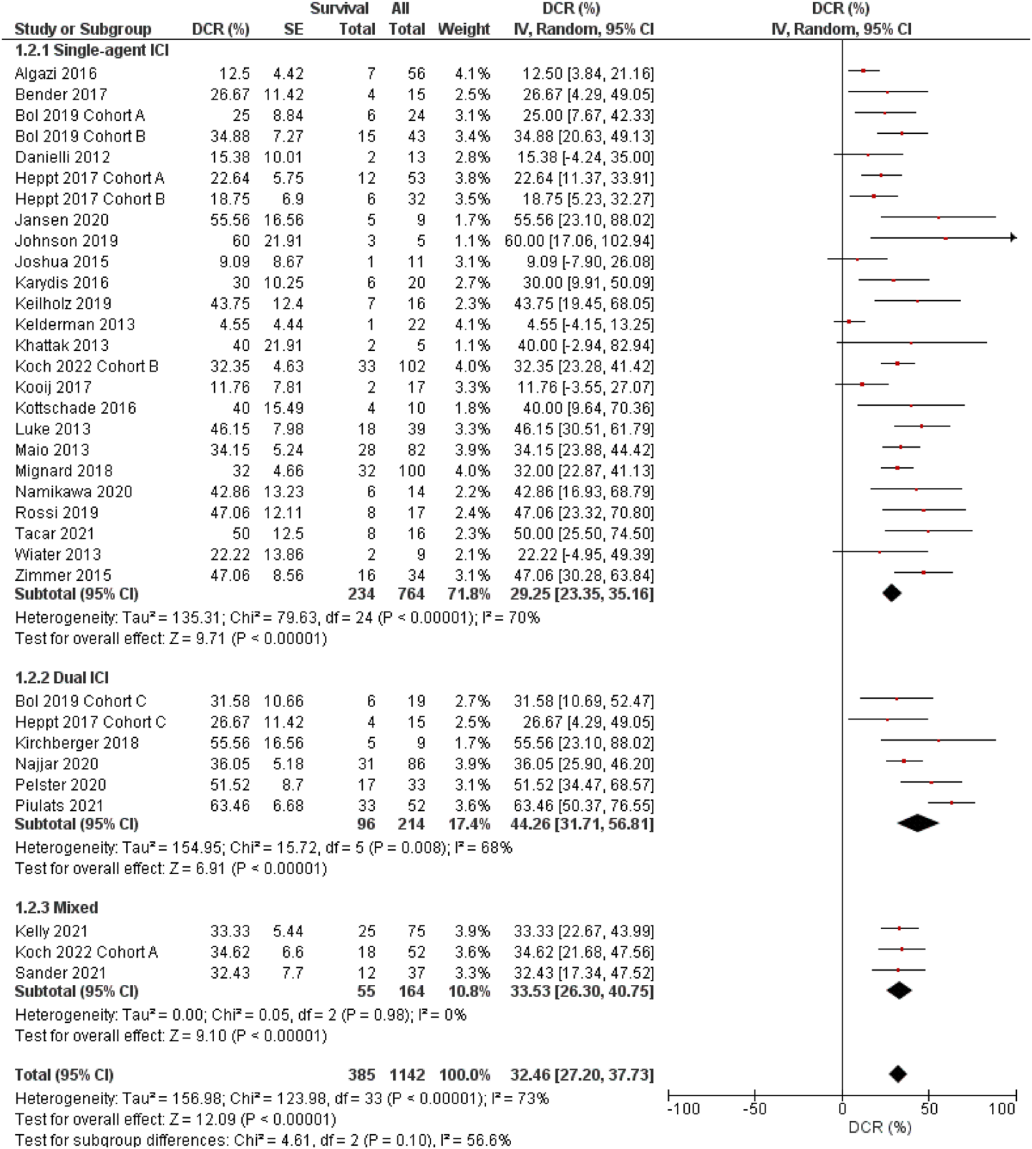
Forest plots for disease control rate (DCR, %) (random-effect model). *IV* inverse variance, *SE* standard error, *CI* confidence interval, *ICI* immune checkpoint inhibitor, mixed a study that collectively analyzed patients with single- and double-ICI regimens. Subgroup comparison single versus dual-agent ICIs, P = 0.03.

The estimated DCR from 34 cohorts was 32.5% (95% CI, 27.2–37.7%; I^2^ = 73%, P for subgroup heterogeneity < 0.001; Fig. 2). Compared to ICI monotherapy (29.3%, 95% CI 23.4–35.2; I^2^ = 70%, P for subgroup heterogeneity < 0.001), dual ICI (44.3%, 95% CI 31.7–56.8%; I^2^ = 68%, P for heterogeneity = 0.008) was effective in more patients with metastatic uveal melanoma (subgroup comparison single-agent versus dual ICIs, P = 0.03, Fig. 2).

### Median progression-free survival

**Figure 3:**
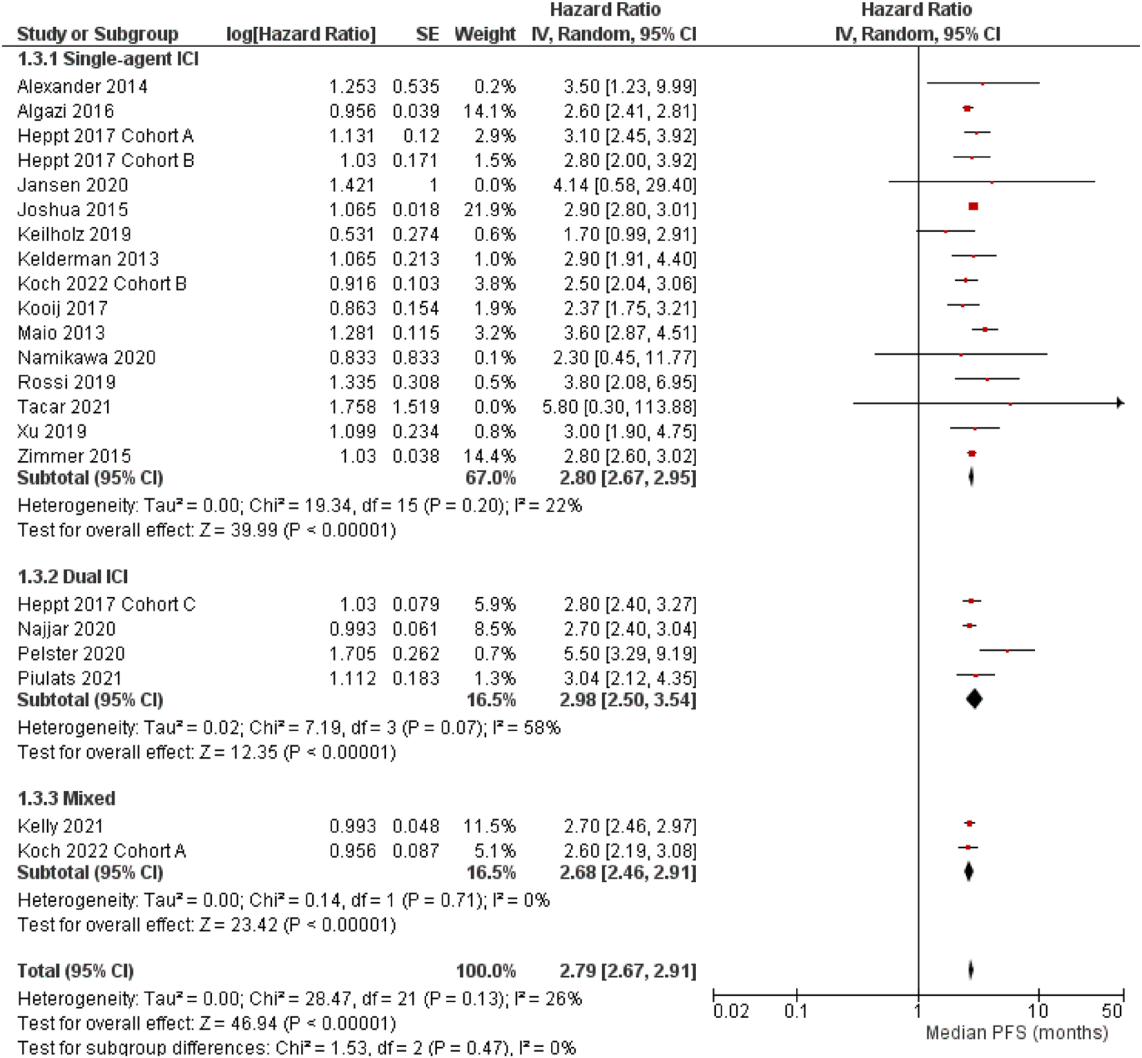
Forest plots for progression-free survival (PFS, months) (random-effect model). *IV* inverse variance, *SE* standard error, CI, confidence interval, *ICI* immune checkpoint inhibitor, mixed a study that collectively analyzed patients with single- and dual-ICI regimens. Subgroup comparison single-agent versus dual ICIs, P = 0.52.

Median PFS data were available for 22 cohorts. Half (n = 11) of these studies reported similar median PFS in the narrow range of 2.6–3.0 months with a precise 95% CI (Fig. 3). For instance, Joshua et al. reported a median PFS of 2.9 (95% CI 2.8–3.0 months) ^33^. The Kaplan–Meier curve reveals a sudden nearly vertical decline in the PFS rate of approximately 2.9 months ^33^.

A meta-analysis incorporating 22 cohorts suggested that the median PFS was 2.8 months (95% CI 2.7–2.9 months, Fig. 3). Neither the cohort (I^2^ = 26%, P for heterogeneity = 0.13) nor subgroup-level heterogeneity (I^2^ = 0%, P for heterogeneity = 0.47) was significant. The aggregated PFS from the single-agent ICI (2.8 months, 95% CI 2.7–3.0; I^2^ = 22%, P for heterogeneity = 0.2) and that from the dual ICI (3.0 months, 95% CI 2.5–3.5; I^2^ = 58%, P for heterogeneity = 0.07) were compatible (subgroup comparison single-agent versus dual ICIs, P = 0.52).

### Median overall survival

**Figure 4:**
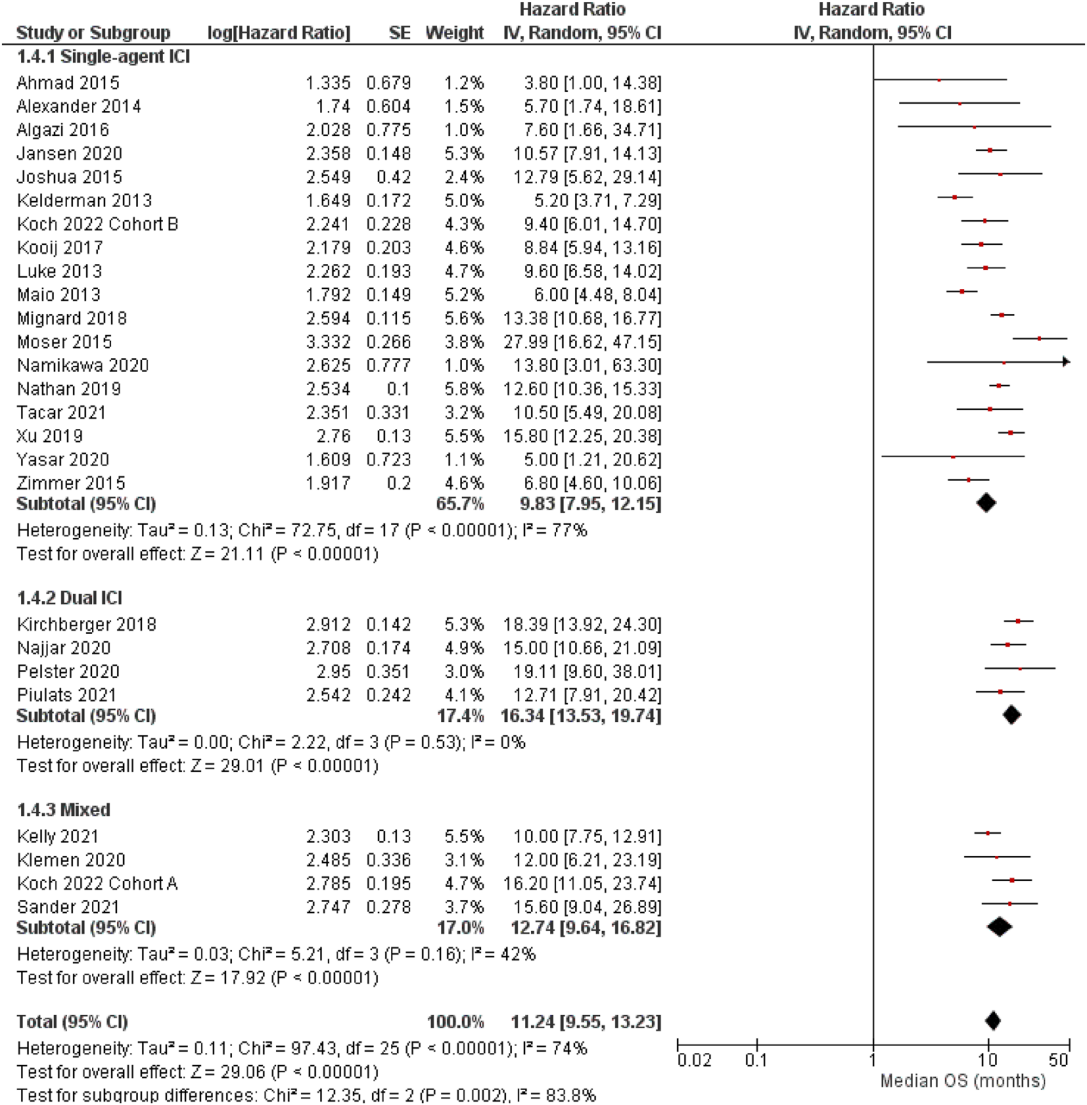
Forest plots for overall survival (OS, months) (random-effect model). *IV* inverse variance, *SE* standard error, *CI* confidence interval, *ICI* immune checkpoint inhibitor, mixed a study that collectively analyzed patients with single- and dual-ICI regimens. Subgroup comparison single-agent versus dual ICIs, P < 0.001.

In contrast to the median PFS, the cohort-level median OS greatly varied from 3.8 to 28.0 months (Fig. 4). According to a random-model meta-analysis, the aggregated median OS among patients with metastatic ocular melanoma was 11.2 months (95% CI 9.6–13.2 months; I^2^ = 74%, P for heterogeneity < 0.001). The patients treated using dual ICI (16.3 months, 95% CI 13.5–19.7 months; I^2^ = 0%, P for heterogeneity = 0.53) survived longer (subgroup comparison single-agent versus dual ICIs, P < 0.001) than those treated using single-agent ICI (9.8 months, 95% CI 8.0–12.2 months; I^2^ = 77%, P for heterogeneity < 0.001).

### Funnel plot

Funnel plots for ORR, DCR, median PFS, and median OS denied clear publication bias (Supplementary Fig. 2).

### Sensitivity analysis

Sensitivity analysis based on a fixed-effect model for ORR, DCR, median PFS, and median OS are illustrated in Supplementary Figs. 3–6. The results indicate the same trend as for the main analysis by using the random-effect model.

## Discussion

We performed a systematic review and meta-analysis to focus on patients with metastatic uveal melanoma who were treated with ICIs. The key strengths of our study include the large number of included studies despite the rarity of metastatic uveal melanoma and a straightforward clinical question related to anti-cancer medications. We demonstrated the following key efficacy outcomes: ORR = 5.6%, DCR = 32.5%, PFS = 2.8 months, and OS = 11.2 months. No systematic review has compared the efficacy of mono- and dual-ICI regimens in the treatment of metastatic uveal melanoma. Our data clarified that the efficacy of ICI monotherapy and dual therapy differed, suggesting that ORR with single-agent ICI and dual ICIs should be evaluated separately. In addition, our subgroup analyses clarified the superiority of dual ICI over single-agent ICI for the treatment.

ICI treatment for metastatic uveal melanoma has been evaluated in numerous small uncontrolled trials and observational studies. In a meta-analysis conducted in 2019, Rantala et al. compared the OS of patients treated with different strategies by combining individual patient-level data of 2,494 cases from 78 articles published between 1980 and 2017, although those were mainly conducted during the time before ICI became available^3^. According to Rantala et al., the median OS after ICI was inferior to that after conventional chemotherapy (hazard ratio 1.13, 95% CI 1.06–1.20, P < 0.0001). They included nine ICI-related studies, which all used ICI monotherapy.

However, single- and dual-agent ICI regimens have recently been regarded as different strategies for various malignancies^17^. The nivolumab plus ipilimumab, the most frequently selected dual ICI therapy, is considered first-line therapy for renal cell carcinoma^12^, non-small cell lung cancer^13^, esophageal cancers^14^, colorectal cancer^15^, and pleural mesothelioma^16^. Dual ICI therapy led to better clinical outcomes in cases with cutaneous melanoma and is a better strategy than ICI monotherapy^60,61^. In a systematic review, Pradeep et al. aggregated data from nine randomized controlled trials that compared “nivolumab plus ipilimumab” and “nivolumab or ipilimumab” for advanced cutaneous melanoma^60^. Pradeep et al. showed that the dual ICI regimen resulted in longer OS (hazard ratio = 0.65, 95% CI 0.53–0.79, P < 0.0001), longer PFS (hazard ratio = 0.48, 95% CI 0.38–0.60, P < 0.0001), and higher ORR (relative risk = 2.15, 95% CI 1.63–2.84, P < 0.001) for cutaneous melanoma. A recent review of systematic treatment for metastatic uveal melanoma by Petzold et al. reported the median OS of anti-PD-1/PD-L1 antibodies (10.9 months, 95% confidence interval (CI): 9.8–13.4), anti-CTLA4 antibodies (7.8 months, 95% CI: 6.8–9.3), and dual-ICIs (15.7 months, 95% CI: 14.4–17.9), demonstrating a superior efficacy of dual-ICIs when compared with that with single ICIs, which is consistent with the results of the present study^62^. In addition, although limited to HLA-A*02:01-positive patients, this study demonstrated the longest median OS in tebentafusp (22.4 months, 95% CI: 19.9–29.6)^62^.

Uveal and cutaneous melanomas originate from melanocytes and have overlapping risk factors. However, both melanomas have major biological inconsistencies. From a clinical point of view, uveal melanoma frequently causes liver metastasis, whereas cutaneous disease does not. Local growth factors, chemokines, and adhesion molecules may facilitate uveal melanoma cell engraftment to the liver^6^. Regardless of these differences between cutaneous and uveal melanomas, the results from our analysis (Figs. 1, 2, and 4) and that of Pradeep et al*.* demonstrated the advantage of the combined ICI regimen for melanoma^60^. CTLA-4 and PD-1 activate immune reactions to tumor cells by blocking different pathways; therefore, dual administration of both ICIs may precipitate powerful anti-tumor reactions^63^. Inhibiting only one pathway can lead to upregulation of the other mechanism; however, combined CTLA-4 and PD-1 can simultaneously downregulate both pathways^60^.

The dual-ICI regimen may prompt more frequent immune-related adverse events (irAEs) than single-agent ICI. Based on previous systematic reviews, pooled frequencies of the key AE indicators caused by dual-ICI are as follows: any AEs, 77.8–81.3%; grade ≥ 3 AEs, 29.3–32.7%; serious AEs, 32.7–34.9%; AE leading to discontinuation, 13.3–28.3%; treatment-related deaths, 0.7–1.0%^64,65^. Considering the increased risk of AEs associated with the combined use of ICI, it is crucial to carefully deliberate and adopt a personalized approach when determining the treatment strategy for uveal melanoma. This approach aims to minimize the development of potential complications in the management of uveal melanoma.

The current study has some limitations. First, a head-to-head regimen comparison was not performed. Second, strong heterogeneities in some outcomes made it challenging to interpret the results. Third, our analysis could not detect a difference in median PFS between single-agent and combined ICIs. Several studies have reported a median PFS of approximately 3 months. These studies may have detected the progressive disease in numerous cases three months after the first course of administration because imaging evaluations were often scheduled just after four cycles of every three-week treatment. Our meta-analysis could not detect PFS differences; nonetheless, the benefits of dual ICI were consistent in ORR, DCR, and OS. Therefore, we believe that dual ICI therapy is superior in treating metastatic uveal melanoma.

## Conclusions

We have reported a systematic review and meta-analysis with particular interest in ICI and the comparison of single- and dual-agent ICIs for the treatment of metastatic uveal melanoma. Despite receiving tebentafusp approval for HLA-A*02:01-positive patients with uveal melanoma, the ICI regimen continues to remain an important choice for the treatment of this disease. Based on the data of 1,414 cases from the 41 cohorts, the pooled ORR, DCR, and OS were 5.6%, 32.5%, and 11.2 months, respectively. Compared to monotherapy, ICI combination therapy (PD-1 + CTLA-4, nivolumab/pembrolizumab plus ipilimumab) significantly improved these outcomes. Our results inform the expected treatment-related outcomes and suggest a future need for randomized trials regarding the dual ICI strategy for metastatic uveal melanoma.

### Supplementary Information

Supplementary Figures.

Supplementary Table 1.

Supplementary Table 2.

## Data Availability

The data supporting the findings of this study are available from the corresponding author upon reasonable request.
