# Supplementary material for: Immune checkpoint inhibitors for metastatic uveal melanoma: a meta-analysis
Source: Sci Rep. 2024 Apr 3;14:7887. doi: 10.1038/s41598-024-55675-5 (PMC10991441; doi:10.1038/s41598-024-55675-5)
Supplement: Supplementary file 1 — Supplementary Figures. [file 41598_2024_55675_MOESM1_ESM.docx]

**Supplementary Figure 1.** The preferred reporting items for systematic reviews and meta-analysis (PRISMA) diagram for the study selection process.


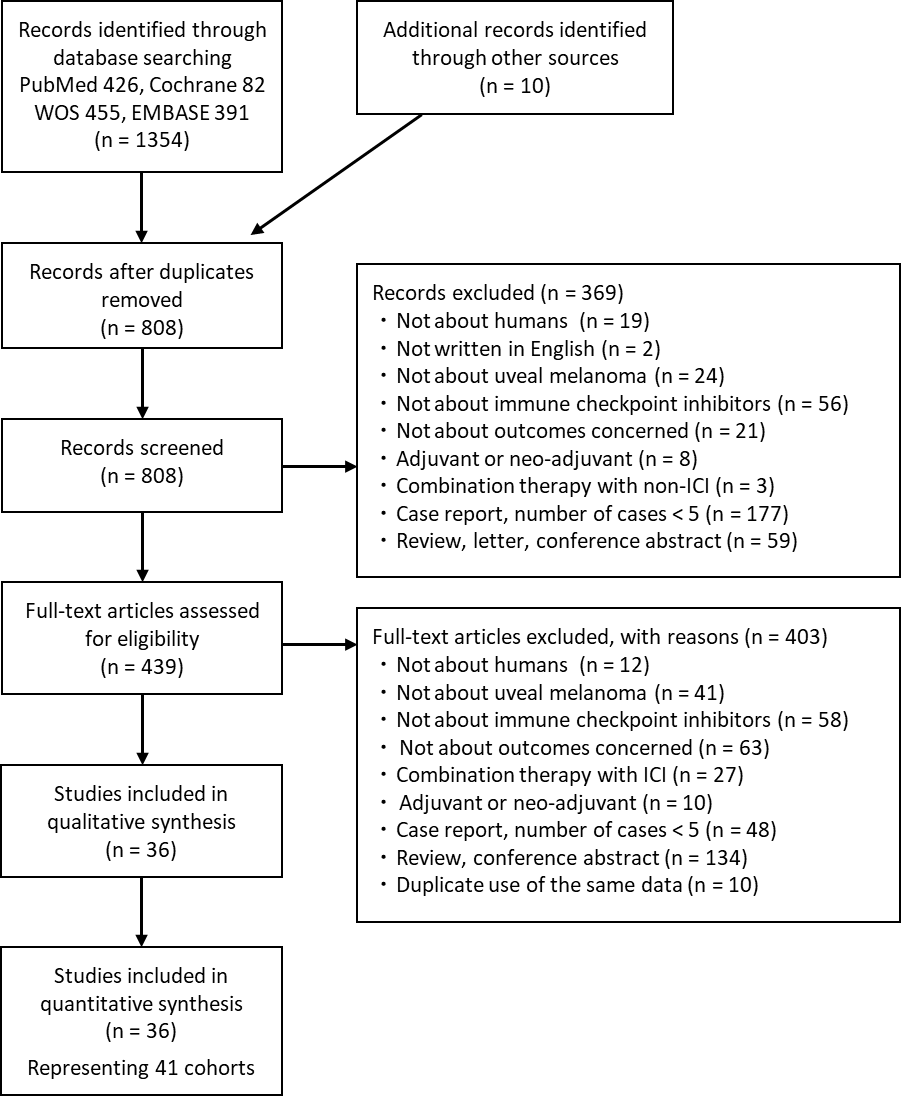


WOS, Web of Science; n, number of articles; ICI, immune checkpoint inhibitor.

**Supplementary Figure 2.** Funnel plots for ORR (%), DCR (%), PFS (months), and OS (months).


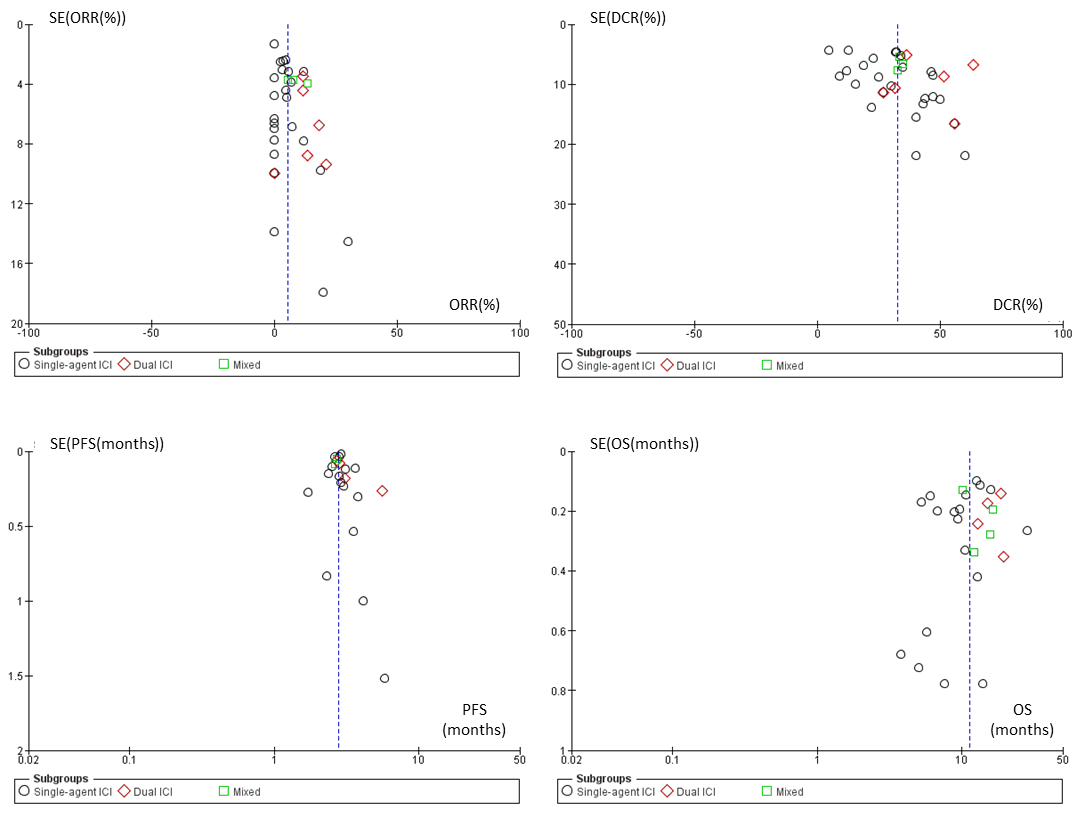


**Supplementary Figure 3.** Forest plots for objective response rate (ORR, %) (fixed-effect model)


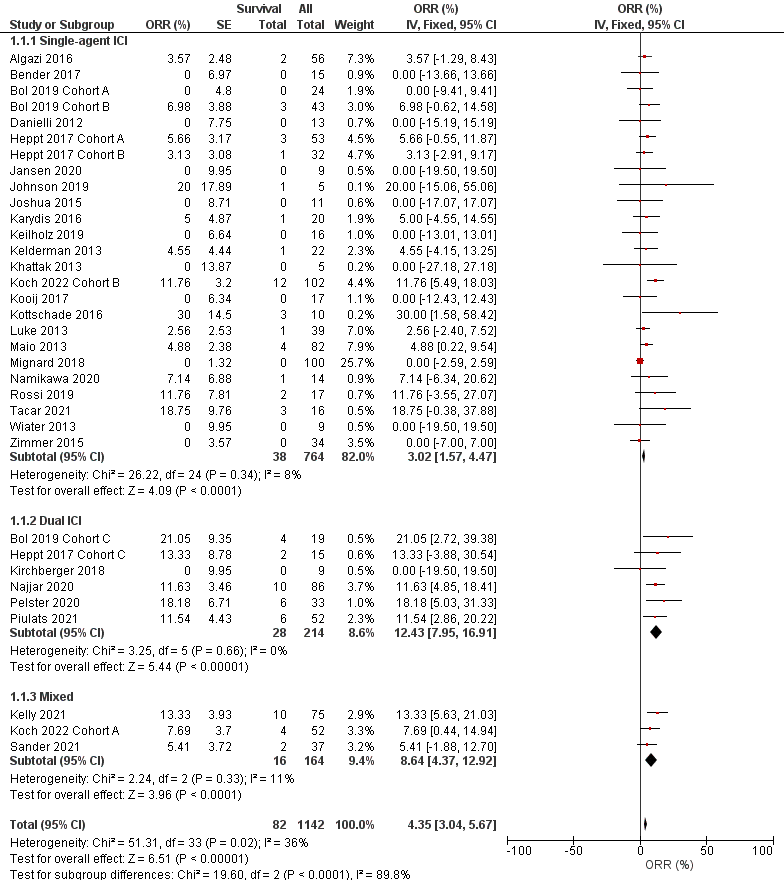


IV, inverse variance; SE, standard error; CI, confidence interval; ICI, immune checkpoint inhibitor; Mixed, a study that collectively analyzed patients with single- and double-ICI regimens.

Subgroup comparison single-agent versus dual ICIs, P < 0.001.

**Supplementary Figure 4.** Forest plots for disease control rate (DCR, %) (fixed-effect model)


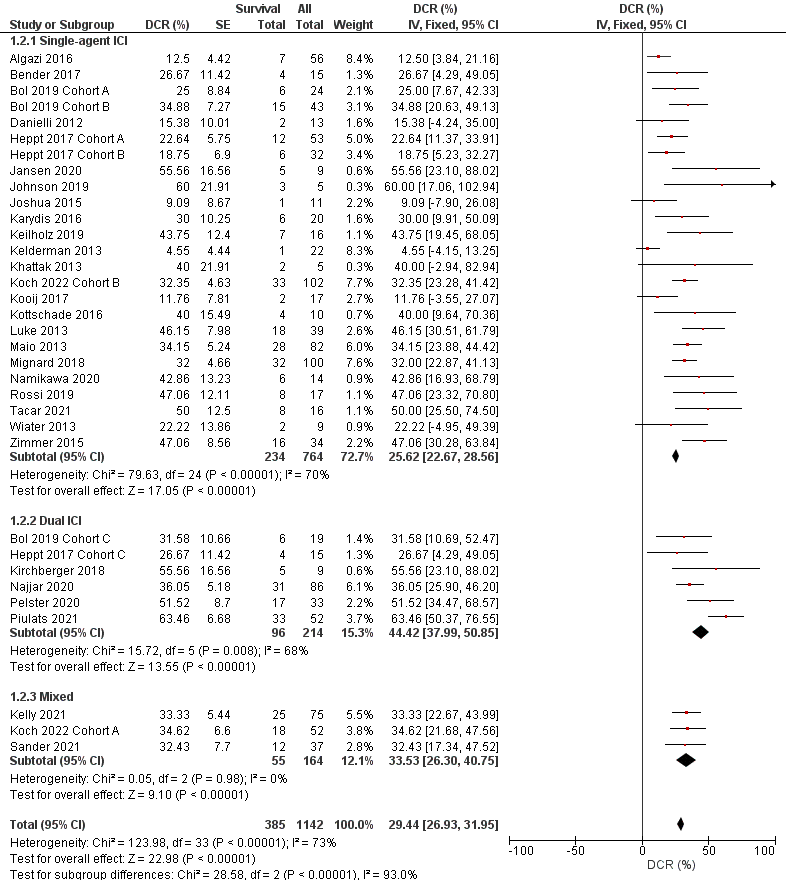


IV, inverse variance; SE, standard error; CI, confidence interval; ICI, immune checkpoint inhibitor; Mixed, a study that collectively analyzed patients with single- and double-ICI regimens.

Subgroup comparison single-agent versus dual ICIs, P < 0.001.

**Supplementary Figure 5.** Forest plots for progression-free survival (PFS, months) (fixed-effect model)


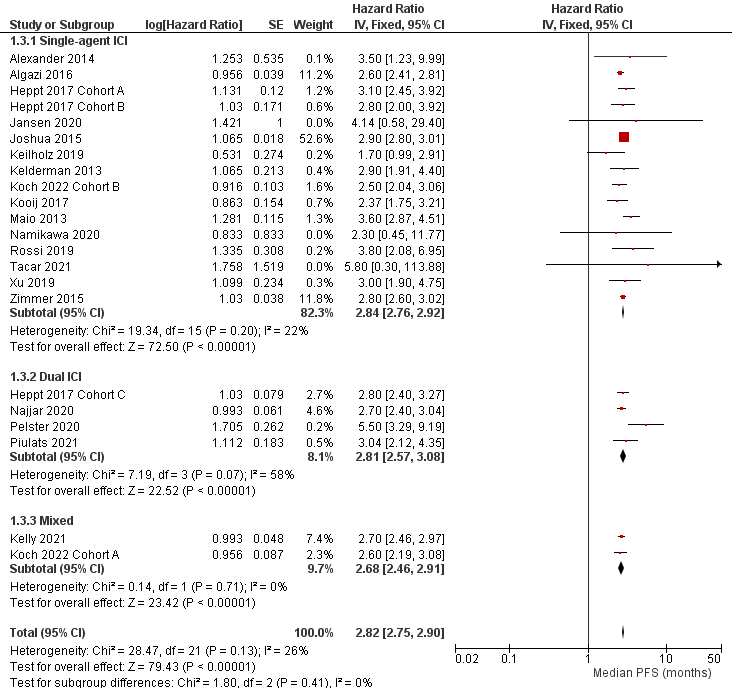


IV, inverse variance; SE, standard error; CI, confidence interval; ICI, immune checkpoint inhibitor; Mixed, a study that collectively analyzed patients with single- and double-ICI regimens.

Subgroup comparison single-agent versus dual ICIs, P = 0.85.

**Supplementary Figure 6.** Forest plots for overall survival (OS, months) (fixed-effect model)


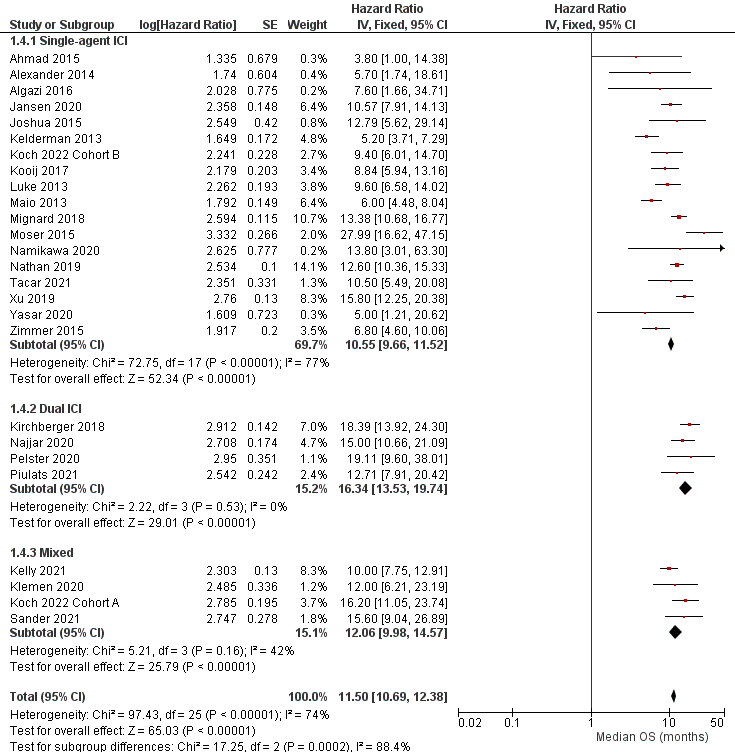


IV, inverse variance; SE, standard error; CI, confidence interval; ICI, immune checkpoint inhibitor; Mixed, a study that collectively analyzed patients with single- and double-ICI regimens.

Subgroup comparison single-agent versus dual ICIs, P < 0.001.
