# Supplementary material for: Immune checkpoint inhibitors for metastatic uveal melanoma: a meta-analysis
Source: Sci Rep. 2024 Apr 3;14:7887. doi: 10.1038/s41598-024-55675-5 (PMC10991441; doi:10.1038/s41598-024-55675-5)
Supplement: Supplementary file 2 — Supplementary Table 1. [file 41598_2024_55675_MOESM2_ESM.docx]

**Supplementary Table 1. Search formulas.**

| Database | Formula | Articles |
| --- | --- | --- |
| PubMed | #1 ICI OR immune checkpoint inhibitor OR immune checkpoint inhibitors OR ICB OR immune checkpoint blockade OR immune checkpoint blockades OR Nivolumab OR Pembrolizumab OR Spartalizumab OR Cemiplimab OR Avelumab OR Atezolizumab OR Durvalumab OR Ipilimumab OR Tremelimumab OR Camrelizumab OR Sintilimab OR Sugemalimab  #2 melanoma  #3 ocular OR eye OR ophthalmological OR intraocular OR uveal OR uvea OR iridal OR iris OR ciliary OR choroidal OR choroid OR choroidea OR chorioidea OR ciliochoroidal  #4 #1 AND #2 AND #3 | 426 |
| Cochrane CENTRAL | #1 ICI OR immune checkpoint inhibitor OR immune checkpoint inhibitors OR ICB OR immune checkpoint blockade OR immune checkpoint blockades OR Nivolumab OR Pembrolizumab OR Spartalizumab OR Cemiplimab OR Avelumab OR Atezolizumab OR Durvalumab OR Ipilimumab OR Tremelimumab OR Camrelizumab OR Sintilimab OR Sugemalimab  #2 melanoma  #3 ocular OR eye OR ophthalmological OR intraocular OR uveal OR uvea OR iridal OR iris OR ciliary OR choroidal OR choroid OR choroidea OR chorioidea OR ciliochoroidal  #4 #1 AND #2 AND #3 [content type = trials] | 82 |
| Web of Science | #1 TS=(ICI OR immune checkpoint inhibitor OR immune checkpoint inhibitors OR ICB OR immune checkpoint blockade OR immune checkpoint blockades OR Nivolumab OR Pembrolizumab OR Spartalizumab OR Cemiplimab OR Avelumab OR Atezolizumab OR Durvalumab OR Ipilimumab OR Tremelimumab OR Camrelizumab OR Sintilimab OR Sugemalimab)  #2 TS=(melanoma)  #3 TS=(ocular OR eye OR ophthalmological OR intraocular OR uveal OR uvea OR iridal OR iris OR ciliary OR choroidal OR choroid OR choroidea OR chorioidea OR ciliochoroidal)  #4 #1 AND #2 AND #3 | 455 |
| EMBASE | Combine the following using AND, Document type = Article/Article in Press  #1 ICI OR immune checkpoint inhibitor OR immune checkpoint inhibitors OR ICB OR immune checkpoint blockade OR immune checkpoint blockades OR Nivolumab OR Pembrolizumab OR Spartalizumab OR Cemiplimab OR Avelumab OR Atezolizumab OR Durvalumab OR Ipilimumab OR Tremelimumab OR Camrelizumab OR Sintilimab OR Sugemalimab [All fields + text]  #2 melanoma [Document title]  #3 ocular OR eye OR ophthalmological OR intraocular OR uveal OR uvea OR iridal OR iris OR ciliary OR choroidal OR choroid OR choroidea OR chorioidea OR ciliochoroidal [All fields + text] | 391 |
